# Developmental Programming: Impact of Prenatal Exposure to Bisphenol A on Senescence and Circadian Mediators in the Liver of Sheep

**DOI:** 10.3390/toxics12010015

**Published:** 2023-12-23

**Authors:** Giuliana Motta, Soundara Viveka Thangaraj, Vasantha Padmanabhan

**Affiliations:** Department of Pediatrics, University of Michigan, Ann Arbor, MI 48105, USA; gmotta@umich.edu (G.M.); svthan@umich.edu (S.V.T.)

**Keywords:** DOHAD, circadian genes, premature senescence, ovine, lipofuscin

## Abstract

Prenatal exposure to endocrine disruptors such as bisphenol A (BPA) plays a critical role in the developmental programming of liver dysfunction that is characteristic of nonalcoholic fatty liver disease (NAFLD). Circadian and aging processes have been implicated in the pathogenesis of NAFLD. We hypothesized that the prenatal BPA-induced fatty-liver phenotype of female sheep is associated with premature hepatic senescence and disruption in circadian clock genes. The expression of circadian rhythm and aging-associated genes, along with other markers of senescence such as telomere length, mitochondrial DNA copy number, and lipofuscin accumulation, were evaluated in the liver tissue of control and prenatal BPA groups. Prenatal BPA exposure significantly elevated the expression of aging-associated genes *GLB1* and *CISD2* and induced large magnitude differences in the expression of other aging genes—*APOE*, *HGF*, *KLOTHO,* and the clock genes *PER2* and *CLOCK*—in the liver; the other senescence markers remained unaffected. Prenatal BPA-programmed aging-related transcriptional changes in the liver may contribute to pathological changes in liver function, elucidating the involvement of aging genes in the pathogenesis of liver steatosis.

## 1. Introduction

Nonalcoholic fatty liver disease (NAFLD) is emerging to be one of the leading liver diseases, with a prevalence of 32.4% worldwide, and is projected to increase to 55.4% by 2040 [1,2]. The recent surge in the incidence of NAFLD, which is considered the hepatic manifestation of metabolic syndrome [3], could be attributed to an increase in metabolic risk factors [4]. According to the concept of the Developmental Origins of Health and Disease (DOHaD) [5], an adverse environment during critical stages of development in early life can induce metabolic changes later in life [6]. Several animal studies have provided evidence in support of the developmental origins of metabolic disorders in offspring [7,8,9]. Along similar lines, the pathogenesis of NAFLD could be linked to adverse developmental conditions [10], including developmental exposure to endocrine-disrupting chemicals (EDCs) that impact hepatic function [11,12,13].

Bisphenol-A (BPA) is an endocrine disruptor found in polycarbonate plastics, epoxy resins, and linings of food containers from where it leaches into food and beverages [14]. There is mounting evidence of the ubiquitous presence of BPA in pregnant women [15], fetuses [16], and human tissues including the fetal liver [17]. Of relevance, exposure to BPA leads to adverse health effects, even at low doses [18], highlighting the risk they pose to public health. In vitro models elucidate the adverse effect of BPA at the materno-fetal interface [19] and human studies show prenatal BPA impacts the expression and epigenetic regulation of xenobiotic metabolizing enzyme genes in the fetal liver [20], which can all contribute to increased susceptibility to diseases later in life. Indeed, epidemiological and animal studies have found that BPA plays a role in developmental programming, and prenatal exposure to BPA has been associated with several metabolic disorders [21]. While epidemiological studies provide the associations between prenatal BPA exposure and metabolic disorders, studies in animal models have established the causal role of BPA in the developmental programming of metabolic disorders [22,23], including hepatic steatosis [24,25].

Relative to the focus of this study, animal models have elucidated the adverse effects of BPA on liver phenotype [26]. A large animal model, sheep is anatomically and physiologically similar to humans, with longer gestation times and a fetal developmental trajectory closer to humans, making it an ideal human biomedical model [27]. Their precocious nature improves the translational value of studies carried out in sheep in the areas of human inflammatory diseases [28], neurological disorders [29], reproductive disorders [30], intrauterine growth restriction [31], and cardiac dysfunction [32]. The sheep model has also been extensively used to understand the developmental origin of metabolic diseases and the critical window of susceptibility for the development of adult metabolic perturbations has been identified as days 30–90 of gestation, using a native steroid [33]. This framework on metabolic susceptibility in sheep offers an advantage in testing the effects of prenatal BPA exposure on metabolic parameters [34,35]. Prior studies using the precocial sheep model show that prenatal BPA exposure leads to increased hepatic oxidative stress and lipid accumulation [36], peripheral insulin resistance [37], and hepatic transcriptome changes of relevance to steatosis [38] in offspring. Studies in small animal models also show that prenatal exposure to BPA affects lipid metabolism in the fetal liver [39], affects the liver lipidome [40], and leads to glucose metabolism dysfunction [26] in offspring. Taken together, these findings suggest that prenatal BPA exposure induces liver dysfunction through the hepatic programming of insulin resistance and liver steatosis, features critical for the progression of NAFLD [41]. The pathogenesis of NAFLD is a multifactorial process that involves several cellular and molecular pathways [42,43].

The disruption of circadian clocks [44] and cellular senescence [45] are emerging as key players in metabolic disorders such as NAFLD. Hepatic senescence has been associated with the reduction of liver metabolism, potentially leading to hepatic and metabolic disease including NAFLD [46,47]. The role of hepatic senescence, a hallmark of liver aging, in inducing the NAFLD phenotype as a result of prenatal BPA exposure is unknown, although the observed liver dysfunction and increased oxidative stress are consistent with age-related changes [48]. Similarly, disruption of the liver circadian clock is related to liver diseases including NAFLD [49]. Prenatal exposure to BPA also alters the hepatic transcriptome related to circadian rhythm in rats [50], indicative of a role for BPA in disrupting circadian rhythm. Additionally, there also exists a close inter-relationship between aging, circadian rhythm, and metabolism [51], as evidenced by mouse studies that demonstrate the disruption of the circadian rhythm in liver mitochondria due to aging [52]. Aging disrupts the liver’s circadian rhythm [51] and the circadian clock, functions at the crossroads of liver metabolism and aging [53], warranting studies that investigate both these aspects. Considering the fact that prenatal BPA exposure has an impact on liver phenotype and the potential involvement of circadian and senescence genes on the liver phenotype, we hypothesize that the prenatal BPA-induced fatty-liver phenotype of female sheep is associated with premature hepatic senescence and a disruption of circadian clock genes.

## 2. Materials and Methods

### 2.1. Animals

All animal procedures were conducted at the University of Michigan Sheep Research Facility (Ann Arbor, MI, USA) and were approved by the Institutional Animal Care and Use Committee of the University of Michigan in keeping with the National Institutes of Health’s Guide for the Care and Use of Laboratory Animals [54]. The Suffolk breed of sheep was used, and their maintenance, breeding, and lambing were performed as described earlier [55]. The animals were housed together and were fed a maintenance diet to prevent obesity.

### 2.2. Prenatal BPA Treatments

Prenatal BPA treatments have been described earlier [36]. Briefly, pregnant sheep were randomly assigned to control (n = 8) and BPA (n = 11) treatment groups. Control ewes were given the vehicle (corn oil), and BPA-treated ewes were given 0.5 (environmental exposure level) mg/kg of BPA (purity ≥ 99%, cat. No. 239658: Aldrich Chemical, Milwaukee, WI, USA) dissolved in corn oil, administered daily through subcutaneous injections during the sexually dimorphic window of gestational day 30 to day 90 of the 147-day gestation period. Humans are primarily exposed to BPA through the oral route but transdermal exposure [56,57] and exposure via inhalation [58,59,60] have also been reported. Prior studies have also confirmed the absence of an effect of the oral or subcutaneous routes of administration on plasma BPA levels in neonate mice [61]. Additionally, oral administration of BPA requires some form of restraint leading to the potential for adverse stress effects, making it an impractical route in a large animal model.

For this study, we used 5 control (the other 3 gave birth only to male offspring) and eleven 0.5 mg/kg/day BPA-treated female offspring. In the case of twin pregnancies, only one randomly selected female offspring from each mother was used for the study. The choice of female offspring is based on our previous phenotyping investigations using this sex, which showed lipid accumulation in the liver following prenatal BPA treatment. The administration of 0.5 mg/kg/day of BPA to the pregnant ewes produced umbilical arterial levels of ~2.6 ng/mL of free BPA on day 90 of fetal life [62], which reflects the range of free BPA (<LOD—52.26 ng/mL) found at mid-gestation in a human cohort study [63], thus reflecting environmental exposure levels. All lambs used in this study were females, weaned at ~8 weeks of age and maintained on a diet of 0.64 kg of corn, 0.64 kg hay·lamb^−1^·day^−1^, and 0.014 kg of supplement (36% crude protein) to avoid the development of obesity.

### 2.3. Tissue Collection

**Figure 1:**
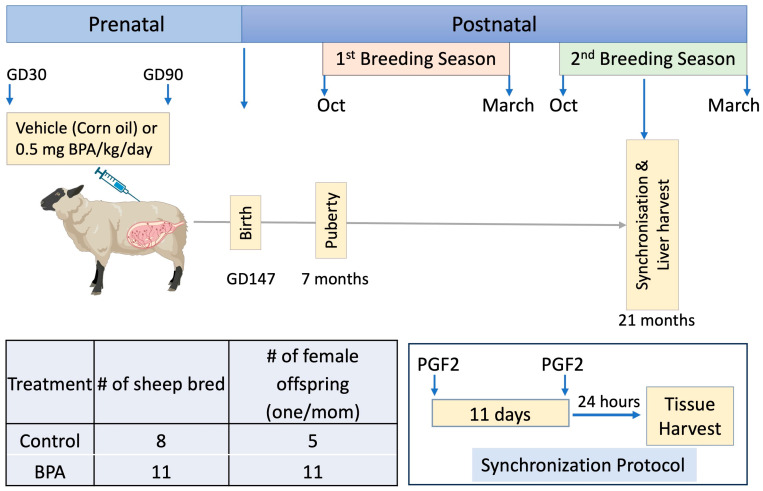
**Summary of experimental design.** (**Top panel**): Schematic showing prenatal treatment (daily subcutaneous injections of vehicle (corn oil) or 0.5 mg/kg BPA in corn oil) from gestational days (GD) 30–90 (term 147 days) and timing of liver harvest from reproductively mature adult 21-month-old female offspring following cycle synchronization. The fatty liver phenotype of BPA-treated females at this age has been previously published [36]. (**Bottom panel**): Treatment groups, number of animals bred, and the number of female offspring studied are shown on the left (note 3 controls only provided male offspring), and the synchronization protocol on the right. Cycle synchronization involved 2 injections of prostaglandin F2 (PGF2) given 11 days apart followed by euthanasia 24 h after the 2nd PGF 2 injection and collection of the liver during the synchronized follicular phase. # denotes the number of animals used.

The focus of this study was to assess the contribution of circadian and senescent genes in the liver to the fatty liver phenotype already characterized at 21 months of age in prenatal BPA-treated reproductively mature adult females [36,37]. The study design is presented in Figure 1. Archived tissues collected from adult females at ~21 months of age (second breeding season) after 48 h of fasting [36] were used in this study. To avoid getting the female pregnant, male offspring were not maintained. Since cyclic changes in steroid hormone levels can influence the circadian rhythm genes [64,65], tissues were harvested after synchronizing cycles with 2 doses of prostaglandin injections (PGF2α, 10 mg, i.m.; Lutalyse, Pfizer Animal Health, Florham Park, NJ, USA), administered 11 days apart. Animals were euthanized by barbiturate overdose (Fatal Plus; Vortech Pharmaceuticals, Dearborn, MI, USA) 24 h after the second dose of prostaglandin, during the late follicular phase. Flash-frozen (stored at −80 °C) and formalin-fixed and paraffin-embedded liver tissue collected from the tip of the left lobe were both used in this study.

### 2.4. RT-PCR

Cellular senescence, one of the hallmarks of aging in the liver [66], was assessed by measuring the expression of genes involved in the aging process, along with measuring mitochondrial DNA copy number and relative telomere length.

#### 2.4.1. mRNA Expression of Circadian Genes and Aging Genes

RNA from 45 mg of frozen liver tissue was extracted using Trizol reagent (Life Technologies, Carlsbad, CA, USA), as per the manufacturer’s instructions. Using a SuperScript VILO kit (Thermo Fisher Scientific, Waltham, MA, USA), 1000 ng of RNA was reverse transcribed, as per the manufacturer’s instructions. Primers were retrieved from the previous literature or designed using Primer BLAST ^®^ (NIH), and the primer sequences are indicated in Supplementary Table S1. Gene expression was analyzed using SYBRgreen-based real-time RT-PCR on the ABI StepOnePlus™ Real-Time PCR System (Thermo Fisher Scientific, Waltham, MA). The cycling conditions used were enzyme activation at 95 °C for 2 min, followed by 40 cycles of denaturation at 95 °C for 15 s and primer annealing at 60 °C for 1 min. A melt curve analysis was performed at the end of the amplification. The relative amount of each transcript was estimated by the ΔΔCT method, using GAPDH as the endogenous reference gene. The reactions were carried out in triplicates.

#### 2.4.2. Mitochondrial DNA Copy Number

A PCR-based analysis of the mitochondrial DNA copy number was conducted as described earlier [67]. DNA was extracted from 45 mg of frozen liver tissue using DNAzol reagent (Molecular Research Center, Inc., Cincinnati, OH), as per the manufacturer’s instructions. For this analysis, a gene encoded by mitochondrial DNA (*CYTB*) was compared to a gene encoded by genomic DNA using GAPDH primers [67]. Samples were analyzed using SYBRgreen-based PCR on the ABI StepOnePlus™ Real-Time PCR System using the cycling conditions described earlier [68]. A melt curve analysis was performed at the end of the amplification. Genes were compared using the following equations: ΔCT = (nucDNA CT − mtDNA CT) and relative mitochondrial DNA content was estimated as 2 × 2^ΔCT^. The reactions were carried out in triplicates.

#### 2.4.3. Telomere Length Assay

Telomere length was measured by a PCR-based method using DNA as described earlier [69]. The sequences of primers coding for telomeres (*TELGC*) and a single copy reference gene (*GDF8*) are shown in Supplementary Table S1. Telomere and reference gene reactions were run on the same plate in separate wells with primer concentrations of 900 nM and 500 nM, respectively, in a total reaction volume of 15 μL with 1 ng DNA. Samples were analyzed using SYBRgreen-based PCR on the ABI StepOnePlus™ Real-Time PCR System using the cycling conditions described by Froy et al. [70] as follows: enzyme activation at 95 °C for 10 min, 50 cycles of denaturation at 95 °C for 15 s, primer annealing at 58 °C for 30 s, and signal acquisition at 72 °C for 30 s. This was followed by a melting curve analysis of 95 °C for 1 min, 58 °C for 30 s, 0.11 °C/s to 95 °C, and finally 40 °C for 10 s. DNA from all the control samples were pooled to create a calibrator sample that was used in every plate run to account for plate-to-plate variation. The single copy *GDF8* (G) gene was used to normalize the telomere (T) data by determining the (T/G) ratio for each sample. The relative telomere length was determined by the factor by which the T/G ratio of the samples differed from the calibrator sample. The reactions were carried out in triplicates.

### 2.5. Lipofuscin Staining

Sudan Black B (SBB) staining of the lipofuscin pigment, a marker of replicative and stress-induced senescence [71], was visualized in the FFPE liver section. SBB staining solution was prepared as outlined by Georgakopoulou et al. [71], by dissolving 0.7 g of SBB in 70% ethanol, filtered and stored in an airtight container. FFPE sections were dewaxed in xylene, dehydrated until 70% ethanol, and immersed in SBB solution for 60 min. The tissues were washed twice in 70% ethanol, six changes of distilled water, counterstained with 0.1% Nuclear Fast Red for 5 min, and mounted with glycerol gelatin slide-mounting medium (Sigma-Aldrich, St. Louis, MO, USA). The slides were dried and imaged at 40x the same day, as the Nuclear Fast Red counterstain diffused into the aqueous mounting medium on storage. The number of cells showing lipofuscin were counted using ImageJ (v1.54b) from 10 fields/section and the average was calculated from three technical replicates—three sections 50 μm apart for each animal. Sections from 8 control (includes 3 additional controls from another cohort treated the same way as the controls used in this cohort) and 7 prenatal BPA animals were used for the staining.

### 2.6. Statistical Analysis

The outliers were identified using the ROUT method and the data were checked for normality using the Shapiro–Wilk normality test. Data that followed normal distribution were analyzed by a two-tailed Student’s *t*-test, while a Mann–Whitney U test was used to analyze data without a normal distribution using Prism v10.1 (GraphPad, La Jolle, CA, USA). All significance was set at *p* value < 0.05. Additionally, the magnitude of difference between the control and prenatal BPA groups was assessed by Cohen’s effect size analysis, where a Cohen’s d of ≥0.8 represented a large effect size/large magnitude differences and a Cohen’s d of ≥0.5 to 0.8 represented a medium effect size/medium magnitude differences. A large Cohen’s d indicates the mean difference is large compared to the variability and the impact is significant in real-world scenarios and a medium effect size indicates a reasonable overall impact [72]. Graphs were generated using Prism v10.1 software (GraphPad, La Jolle, CA, USA).

## 3. Results

### 3.1. Effect of Prenatal BPA Exposure on Circadian Function

**Figure 2:**
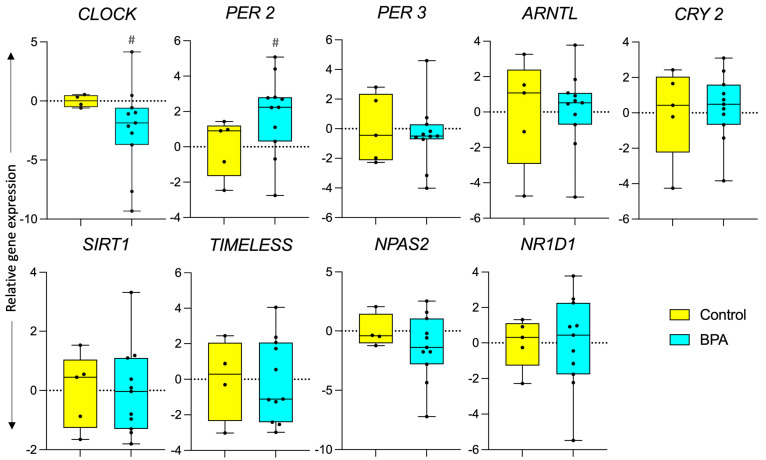
**Effect of prenatal BPA exposure on circadian gene expression.** Box plots for gene expression changes of circadian genes in control (n = 5) and prenatal BPA (n = 11) sheep liver. # represents a large effect size based on Cohen’s d analysis.

Prenatal BPA exposure had no effect on the expression of nine circadian genes, *ARNTL*, *CLOCK*, *CRY2*, *PER2*, *PER3*, *SIRT1*, *NR1D1*, *NPAS2,* and *TIMELESS,* as illustrated in Figure 2. However, there was a large effect size decrease in the *CLOCK* (Cohen’s d = 1.26) gene and an increase in the *PER2* (Cohen’s d = 1.01) gene. 

### 3.2. Effect of Prenatal BPA Exposure on Markers of Longevity and Senescence

**Figure 3:**
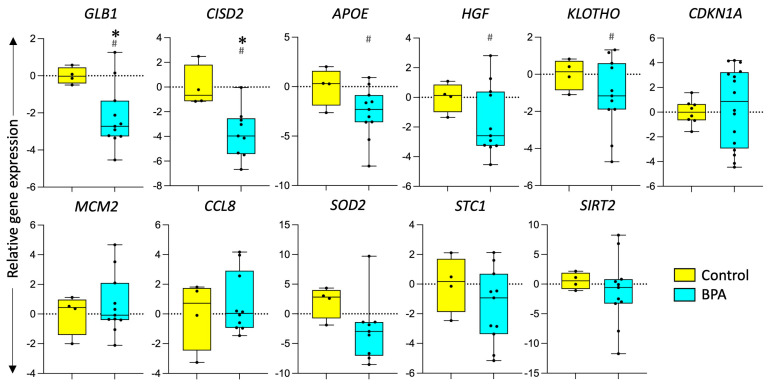
**Effect of prenatal BPA exposure on markers of longevity and senescence.** Box plots for the gene expression of aging-associated genes in control (n = 5) and prenatal BPA (n = 11) sheep. * represents a significant difference (*p* < 0.05) using a Student’s *t*-test, # represents a large effect size using Cohen’s d analysis.

**Figure 4:**
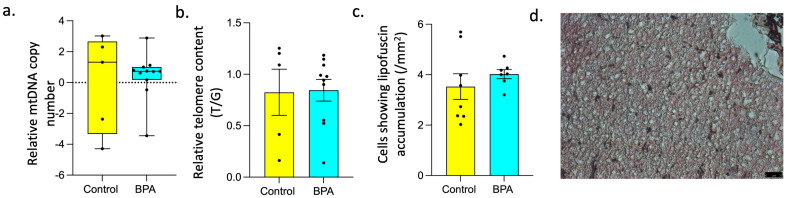
**Effect of prenatal BPA exposure on Mitochondrial DNA copy number, Relative Telomere content, and Senescence.** (**a**) Box plots for mitochondrial DNA copy number in control (n = 5) and prenatal BPA sheep liver (n = 11), (**b**) Bar graph showing relative telomere content in control (n = 5) and prenatal BPA sheep liver (n = 11), (**c**) Bar graph showing lipofuscin granule distribution in control (n = 8) and prenatal BPA (n = 7) sheep liver, (**d**) Representative image showing lipofuscin granules stained by Sudan Black B in liver cells at 40× magnification.

Prenatal BPA exposure increased expression of *GLB1* (*p* = 0.02) and *CISD2* (*p* = 0.01). Expression of other genes involved in the aging process such as *CDKN1A*, *SIRT2*, *MCM2*, *APOE*, *STC1*, *KLOTHO*, *HGF*, *CISD2*, *SOD2,* and *CCL8* did not significantly differ between the control and prenatal BPA groups, as illustrated in Figure 3. The expression of *APOE* (Cohen’s d = 1.23), *HGF* (Cohen’s d = 1.03), and *KLOTHO* (Cohen’s d = 0.9) showed a large magnitude increase in liver tissue in response to prenatal BPA exposure. The telomere length, a marker of biological aging [73], was also not significantly affected in response to prenatal BPA exposure. Similarly, mitochondrial DNA copy number (mtDNA-CN), associated with several aging-related diseases [74,75] and lipofuscin accumulation, was not affected by prenatal BPA exposure and is represented in Figure 4.

**Figure 5:**
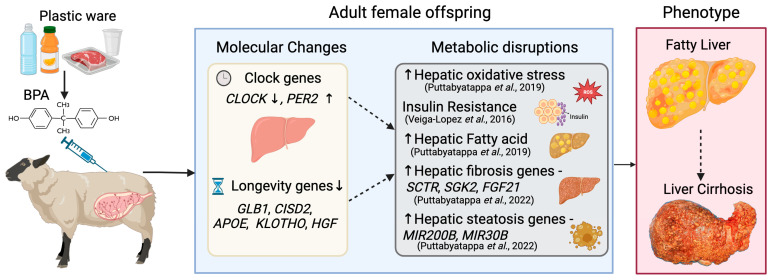
**Graphical Summary.** Summary of Circadian and Aging-related changes programmed by prenatal BPA exposure in the liver, their relationship with previous findings in a sheep model, and potential contribution to the NAFLD phenotype, which is characterized by fatty liver and leads to liver cirrhosis. Our previous publications detailing phenotypic outcomes have been indicated in parenthesis-Puttabyatappa et al., 2019 [36], Veiga-Lopez et al., 2016 [37] and Puttabyatappa et al., 2022 [38].

Figure 5 provides a summary of the findings of this study in the context of metabolic disruptions identified in previous studies and the overall contribution of prenatal BPA exposure to a NAFLD phenotype.

## 4. Discussion

Findings from this study demonstrated that prenatal BPA exposure influenced aging-associated genes and circadian gene expression (marginally) in the liver of the female offspring. The potential contribution of these changes in increasing oxidative stress, lipid accumulation, and insulin resistance, previously reported in the liver of female sheep prenatally exposed to BPA [36,37], is discussed below.

### 4.1. Impact of Prenatal BPA on Circadian Genes

The interplay between the master circadian regulator, the suprachiasmatic nucleus (SCN) in the hypothalamus, and the peripheral circadian clocks in the liver play a vital role in energy homeostasis [76]. The clock genes are expressed in a circadian manner, showing cellular rhythmicity in sheep liver [77,78] and modulating glucose homeostasis [79,80]. *PER2* plays an important role in liver diurnal metabolism, regulation of lipid metabolism [81], and glucose homeostasis [82], and exacerbates nonalcoholic steatohepatitis, a progressive form of NAFLD [83]. The large magnitude increases in *PER2* and decreases in *CLOCK* gene expression evident in prenatal BPA-treated sheep liver (this study) is in agreement with findings of gestational exposure to BPA affecting circadian gene expression in the rat liver [50]. The increasing trend of *PER2* expression, seen in our study, may have contributed to insulin resistance, increased oxidative stress, and ectopic lipid accumulation evidenced earlier in the liver of this sheep model of prenatal BPA exposure [36,37]. In support of this premise, an increase in *PER2* was found to be associated with insulin resistance in the mouse liver [84] and other conditions of increased oxidative stress such as fasting [85]. On the contrary, increased levels of the PER2 protein were associated with reduced fat accumulation in mouse hepatocytes [86]. These differences are a likely function of species differences, or our studies relating to mRNA expression as opposed to the mouse study exploring protein levels. Findings from other studies showing PER protein accumulation repressing *PER2* transcription [87] suggest that the elevated *PER2* transcript level evident in our study is a likely response to decreased PER2 protein expression.

The *CLOCK* gene is the master regulator of circadian rhythms and its disruption impairs metabolic homeostasis [88], leading to hyperlipedemia, hepatic steatosis, hyperglycemia, and hypoinsulinemia in mouse models [89]. In addition, human studies have shown genetic variations in the *CLOCK* gene to be associated with NAFLD [90]. Downregulation of the *CLOCK* gene, as seen in our study, is an indication of compromised circadian rhythmicity, suggesting the regulatory impact of prenatal BPA on the circadian regulation of hepatic *CLOCK* transcription. The decrease in *CLOCK* expression may have contributed to the increased hepatic oxidative stress seen in sheep prenatally exposed to BPA [36]. This premise is supported by studies in *CLOCK* mutant mice that show an increase in oxidative stress [91] and the downregulation of the CLOCK protein in other conditions of increased oxidative stress [92]. In the liver, the regulation of circadian genes by *CLOCK* is also carried out by its ortholog, *NPAS2* [93]. The absence of change in *NPAS2* in the present study may explain the absence of change in the expression of the other CLOCK-controlled genes, in spite of the downregulation of *CLOCK*.

### 4.2. Impact of Prenatal BPA on Longevity and Senescence Genes

Increased oxidative stress and lipid accumulation evidenced earlier in the prenatal BPA exposure sheep model [36] have been linked to the aging process [94], particularly senescence [95]. Cellular senescence, one of the hallmarks of aging, contributes to the progression of NAFLD [96], with NAFLD patients showing increased cellular senescence [97]. We have shown the downregulation of the *GLB1* gene, which codes for senescence-associated β-galactosidase (SA-β-gal), in the liver of sheep prenatally exposed to BPA. Elevated levels of *GLB1*, a lysosomal marker of cell senescence, is associated with increased liver fat, NAFLD, aging, and type 2 diabetes mellitus [98,99,100], and the low levels of *GLB1* seen in our study is in contrast to our other markers pointing at accelerated aging. However, SA-β-gal activity is not essential for senescence [101], making it a non-specific marker of cellular senescence [102]. The gene CDGSH iron-sulfur domain 2 (*CISD2*), one of the pro-longevity genes that protects the liver from age-related pathological conditions and is a molecular target for NAFLD treatment [103], is downregulated in response to prenatal BPA in the liver, indicating an aging phenotype. The increase in oxidative stress reported in this model [36] could also be contributed by the lower level of *CISD2*, as *CISD2* haploinsufficiency induces oxidative stress and NAFLD [104]. *CISD2* is also downregulated during normal aging in the mouse liver [105,106]. Prenatal BPA exposure also induced a large effect size decrease in the mRNA of other anti-aging genes—*APOE* [107], *KLOTHO* [108], and the antiapoptotic and antifibrotic gene *HGF* [109]. APOE deficiency is associated with aging-related changes and hyperlipidemia and promotes NAFLD in mice [110,111,112]. The anti-aging protein KLOTHO also regulates insulin signaling [113], lipid metabolism [114], and oxidative stress [115,116]. Recent epidemiological studies show higher levels of plasma KLOTHO in diabetic patients [117,118] while a lower level of KLOTHO is associated with NAFLD [119]. *HGF* plays a critical role in liver metabolism as HGF treatment decreases fasting blood glucose levels and hepatic lipid content in mice fed a high-fat diet [120] and serum HGF is a marker of NAFLD [121]. The decreasing trend observed in the expression of these longevity genes collectively points towards accelerated liver aging and a possible contribution to the increased lipid accumulation, oxidative stress, and insulin resistance seen in the liver of sheep prenatally exposed to BPA [36,37].

### 4.3. Impact of Prenatal BPA on Other Markers of Senescence

Mitochondrial dysfunction seen in states of increased oxidative stress and cellular senescence [122] has been implicated in age-related diseases [123]. Mitochondrial DNA (mtDNA) copy number is a proxy for mitochondrial function [124] and elevated liver mtDNA is associated with NAFLD [125]. Our results indicate an absence of an effect of prenatal BPA exposure on liver mtDNA copy number. Telomere length shortening is another marker of aging that has been linked to oxidative stress [126], impaired lipid metabolism [127], and several diseases [128], including NAFLD [46,129,130]. Liver telomere length was not affected by prenatal BPA exposure. Lipofuscin accumulation, an indicator of cellular senescence [131], was also not observed in the liver of sheep prenatally exposed to BPA.

The directionality of aging-related/longevity genes, coupled with an absence of change in cellular senescence markers in the liver, suggests that exposure to prenatal BPA impacts cellular aging in liver cells that may ultimately progress towards early senescence when the animal ages. While several other aging genes have been identified, their mechanism of action in the aging process is unknown [132]. As such, alternate mechanisms of senescence may have been missed in this study.

### 4.4. Strengths and Limitations

The strength of this study is the use of a precocial, large animal model with a developmental trajectory similar to humans to elucidate the potential effects of prenatal BPA exposure in humans. The clock gene rhythm in the sheep liver is regulated by a photoperiod [77], providing an avenue to explore the role of prenatal BPA on circadian gene expression in a large animal model. Hyperglycemia and hyperinsulinemia induce hepatic steatosis in sheep [133]. Women with polycystic ovary syndrome (PCOS) are known to develop NAFLD [134] and a similar liver phenotype has been illustrated in a sheep model of PCOS by us [135] and others [136], making this a valid model to elucidate the potential effects of prenatal BPA exposure in the context of changes seen in humans. The maternal BPA treatment of 0.5 mg/kg/day produced umbilical arterial levels of ~2.6 ng/mL of free BPA on day 90 of fetal life [62]. These levels are within the range of BPA found in cord blood samples (range of 0.53–4.75 ng/mL) [63,137,138,139,140] from human cohort studies and are relevant to environmental exposure levels. BPA is ubiquitously present in the environment, air, water, soil, animal feed, wildlife, and humans [141,142,143]. While the role of direct BPA exposure in the development of diabetes mellitus and obesity has been explored extensively [144,145], there is a gap in the research on the prenatal effect of BPA on liver pathologies. This study was performed to address causal relationships between prenatal BPA and liver steatosis, as human studies can only point to association and not causality. With the emerging molecular link between senescence and circadian rhythm, this is the first study to look at the prenatal effects of BPA on senescence and circadian clock genes in sheep liver tissue, at a dose relevant to human environmental exposure.

Some of the limitations of this study include the fact that the liver tissue was not harvested according to zeitgeber times, and the time of tissue collection was not synchronized across all the animals due to practical difficulties in working with large animal models. This could have resulted in a shift in expression due to the rhythmicity in the circadian genes. The inter-animal differences could have led to variability in the amplitude of the circadian rhythm between them [146], which would have influenced the circadian gene expression. Post-transcriptional and post-translational modifications play a key role in the rhythmic expression of circadian genes in the liver [147], which was not evaluated in this study. Although several studies have reported Sudan black B staining of lipofuscin as a marker of cellular senescence, it also diffusely stains distributed lipids in cells [148]. Ideally, an assay for β-galactosidase, which is expressed from *GLB1* and is a biochemical marker of senescence, would have been more specific. However, the assay required fresh tissue or cryosections that we did not have from this cohort of animals.

## 5. Conclusions

This study indicates that prenatal exposure to BPA, at a dose of human relevance, leads to changes in the liver of adult female offspring consistent with premature senescence. These changes in senescence genes link to previously reported phenotypic changes in the liver of this model. Further studies need to be performed to address the mechanism of action of these genes on liver function and their role in liver dysfunction. These results assume significance with the increasing prevalence of BPA and metabolic diseases like NAFLD among the general human population.

## Data Availability

The data presented in this study are available on request from the corresponding author.

## Supplementary Materials

The following supporting information can be downloaded at: https://www.mdpi.com/article/10.3390/toxics12010015/s1, Supplementary Table S1: Primer sequences used to determine the expression of circadian genes, longevity/senescence genes, mtDNA copy number and relative telomere length [149,150,151].
