# Supplementary material for: Developmental Programming: Impact of Prenatal Exposure to Bisphenol A on Senescence and Circadian Mediators in the Liver of Sheep
Source: Toxics. 2023 Dec 23;12(1):15. doi: 10.3390/toxics12010015 (PMC10818936; doi:10.3390/toxics12010015)
Supplement: Supplementary file 1 [file toxics-12-00015-s001.zip › toxics-2752656-supplementary.pdf]

Supplementary Table S1: Primer sequences used to determine the expression of circadian genes, longevity/senescence genes, mtDNA copy number and relative telomere length.

**Gene Name**	**Gene Function**	**Forward and Reverse Primers**	**Accession Number**	**Reference**	**Product Length (bp)**
ARNTL	Core Clock gene	5'AATCCACAGCACAGGCTATTT3'<br>3'CGACAAGGCAGCTAAGATTACA5'	NM_001129734.1	[149]	104
CLOCK	Regulates circadian rhythm	5'CAGTCAGTCTCAAGGAAGCG3'<br>3'CTTTGTTGGTGTAGAGGAAGGG5'	NM_001130932	[78]	122
CRY2	Light entrainment of retinal biorhythms	5'GGCTCAACATCGAGCGGAT3'<br>3'CAGATGCCAGCAGACAGAGT5'	NM_001129736	Primer BLAST	74
PER2	Component of the circadian rhythms of locomotor activity, metabolism, and behavior.	5'AAGTCTGAACACCACCTGTC3'<br>3'CTTCAGCTCCTTCAGGGTTT5'	XM_027967088.2	[149]	99
PER3	Component of the circadian rhythms of locomotor activity, metabolism, and behavior.	5'TCTTTCATCATTGGTCGCCA3'<br>3'TGGTGTCTTTGTCATCATTGCT5'	XM_042257313.1	[55]	100
SIRT1	Regulate epigenetic gene silencing	5'TTGGGTACCGAGATGACCTTC3'<br>3'GCATGCGAGGCTCTATCATCT5'	XM_015104377.3	Primer BLAST	94
NR1D1	Negatively regulates the expression of core clock proteins	5'TAACAACAACACAGGTGGCG3'<br>3'ACTGTACAGGGATTCAGGGC5'	XM_004021888	[78]	85
NPAS2	Regulates circadian rhythm	5'ACCAGCACCTGCTAAGAGAAT3'<br>3'CTGCTAAACTGGGGTGTCAG5'	XM_004006132.5	Primer BLAST	106
TIMELESS	Role in cell survival after damage/stress, autoregulates circadian rhythm	5'ACTACCTGAAACGCTTCGCA3'<br>3'TGCAGCATCTTGACCACACA5'	NM_001128153.1	Primer BLAST	112
CDKN1A	Regulates cell cycle	5'TGCCGCTGCCTCTTTGGT3'<br>3'AAAGTCGAAGTTCCATCGCTCT5'	XM_012100423.4	Primer BLAST	108

SIRT2	Regulate epigenetic gene silencing	5'ACCCTTTCCTCGGGATGATG3'<br>3'TTGCATGCTCCTTCCGAACA5'	XM_027978260.2	Primer BLAST	171
MCM2	Initiation of eukaryotic genome replication	5'GAGATCCACCACCGCTTCAA3'<br>3'TCTCGCGGTTCTCTTTGCAC5'	XM_027957871.2	[150]	106
APOE	Catabolism of triglyceride-rich lipoprotein constituents	5'TCGCTGGGACTGCTGATAGA3'<br>3'TGCGATTGGCCAACCTCCTC5'	XM_027978460.2	Primer BLAST	83
STC1	Regulation of renal and intestinal calcium and phosphate transport, cell metabolism	5'AACAGCGCACTGCAGGTTG3'<br>3'TTGACAAATGCTTTTCCCTGAGT5'	XM_004004182.5	Primer BLAST	140
KLOTHO	Regulates metabolism of phosphate, calcium, and vitamin D	5'GACAACTGCATTCAAGTGGACA3'<br>3'GGACATCCCACAGGTAGACG5'	XM_004012279.5	Primer BLAST	70
HGF	Regulate cell growth, cell motility and morphogenesis	5'GGCCAGGTGACCTTTTCTTTG3'<br>3'CTTCTTTTCTTCTGTCCTTCTGC5'	XM_004007806.5	Primer BLAST	166
GLB1	Senescence marker	5'GCGCGTGGTTTGCACAATG3'<br>3'GAGATGTAGCGGAAGGGCTG5'	XM_015102219.3	Primer BLAST	95
CISD2	Role in calcium homeostasis	5'CCGTTCCTCCCAAAGAAAAAGC3'<br>3'CCATCACAGGCAGGAAACGTC5'	XM_004009661.4	Primer BLAST	164
SOD2	Antioxidant enzyme	5'TCAATAAGGAGCAGGGACGC3'<br>3'GCTGCAAGCTGTGTATCGTG5'	NM_001280703.1	[151]	209
CCL8	Role in immunoregulatory and inflammatory processes	5'TGCTCGCTCAGCCAGATTCAG3'<br>3'CCTCCTTGTCCGCTTTGGTC5'	XM_004012469.5	[150]	159
CYTB	Mitochondrial gene	5'ACTCGGAGACCCAGACAACT3'<br>3'TGTAGGG GTGTTCAACTGGC5'	KY662385.1	[67]	296
GAPDH	Housekeeping gene	5'GTCAAGGCAGAGAACGGGAA3'<br>3'GGTTCACGCCCATCACAAAC5'	XM_012166462.1	[67]	232
TELGC	Telomeric DNA	5'ACACTAAGGTTTGGGTTTGGGTTTGGGTTTGGGTTAGTGT3'<br>3'TGTTAGGTATCCCTATCCCTATCCCTATCCCTATCCCTAACA5'		[69]	79

GDF8	Single copy gene	5'TGGAGTTCGTCTTTCCAACC3'<br>3'GGAAGGCAGAGTGATGAAGG5'		[69]	
